# A quality improvement project to increase patient feedback in the psychotherapy department, Tavistock Clinic

**DOI:** 10.1192/bjo.2021.471

**Published:** 2021-06-18

**Authors:** Avgoustina Almyroudi, Alan Baban, Sukhjit Sidhu

**Affiliations:** Tavistock and Portman NHS Trust

## Abstract

**Aims:**

A rigorous and systematic patient feedback system is important for identifying gaps, improving the quality of care and encouraging patient involvement in service delivery. In the Adult Complex Needs Service of the Tavistock Clinic, a tertiary psychotherapy centre, only 5% of patients have provided feedback when requested. This Quality Improvement (QI) project aimed at improving the return rates of the Experience of Service Questionnaire (ESQ) and the CORE Outcome Measure by 10% within a year.

**Method:**

The QI methodology was used to help identify factors contributing to the low response rate, including views amongst stuff about how such feedback, and the method of its delivery, might affect a psychoanalytically-informed treatment. Previously these forms were posted or handed out in person. In the first Plan-Do-Study-Act (PDSA) cycle, the method of distribution was changed by sending out the questionnaires to patients electronically, using an online survey platform. In the second PDSA cycle, the CORE-34 questionnaire was replaced with a shorter version, the CORE-10. This was in order to test our hypothesis that a shorter questionnaire would result in an increase in the response rates.

**Result:**

In the first cycle of change, 197 patients were emailed for both the CORE-34 and ESQ and a total return rate of 31% was achieved. This signified an increase of 26% in the response rate. Overall more ESQ forms were completed (35% uptake) compared to CORE-34 forms (28% uptake). In the second cycle 199 patients were emailed with the CORE-10 and ESQ forms. The response rate was 21% and 18% respectively. Although the response rates decreased slightly in the second PDSA cycle the results indicated that this method of distribution was capturing a greater range of patients who had not previously provided the service with this sort of feedback.

**Conclusion:**

Sending out the outcome measures electronically and adopting shorter versions of the CORE questionnaire increased the feedback response rate significantly, and provided the service with useful data as to patients' experience of their treatment journey here.

